# Cause of the Blending of Twins, and Their Being of the Same Sex

**Published:** 1852-02

**Authors:** Silas Hubbard

**Affiliations:** Buffalo


ART. IV.—*Cause of the Blending of Twins, and their being of the same Sex.* By SILAS HUBBARD, M. D., of Buffalo.

On page 252, vol. 6 of your Journal, I advanced the theory that girls are begotten during the first half, and boys the latter half of the inter-menstrual period, and with this understanding I endeavored to assign a cause for extra-uterine conceptions in the last Sept. No. of your Journal; and now, with the same understanding, I have been induced to search and have observed, so far as I have ascertained, that in all those cases of monstrosities in which individuals adhere together, they are of the same sex. Now I propose to offer the probable reason why they are of the same sex. They are of the same sex because they are begotten at the same time and under the same circumstances. Their respective ova being either impregnated while contiguous in the uterus or while in the Fallopian tube or tubes, or ovary or ovaries, in which latter cases they are either deposited in the uterus together, or at nearly the same time, contiguous to each other, and thus grow together in a very regular way with corresponding parts connected or welded together, which is not likely to occur in cases of superfœtation. The examples I have of united twins being of the same sex, are seven, viz: three respectively in the Castleton, Albany, and Buffalo Medical Museums, three mentioned by Prof. Meigs, in his last edition of obstetrics, and the Siamese Twins. To these a few specimens I have witnessed of animals. It would be useful if physicians would note, and eventually publish, the particulars of every undoubted case tending to prove or disprove any of these theories.

BUFFALO, January, 1852.

